# Influence of fruit drinks with or without lactobacillus Lp299v on the gastrointestinal uptake of paracetamol in man

**DOI:** 10.1186/1756-0500-2-45

**Published:** 2009-03-20

**Authors:** Ulrika Åkerman, Lars Edvinsson

**Affiliations:** 1 Department of Emergency Medicine, Clinical Sciences, University Hospital, Lund, Sweden

## Abstract

**Background:**

Clinical observations have revealed that patients throw up undigested paracetamol tablets several hours following intake of rosehip drink with Lp299v (Proviva). The purpose of this study was to demonstrate if this translates into altered plasma levels of paracetamol in nineteen healthy subjects consuming 200 ml of water, Rose hip drink or Proviva together with 1.5 gram of paracetamol.

**Findings:**

The concentration of paracetamol in plasma increased rapidly when the paracetamol-containing tablets were consumed together with water and after 30 minutes a median level of 90 μmol/l was reached (a 95% confidence interval of 57,161). The corresponding 30 minutes values of paracetamol levels in the presence of rosehip with or without Lp299v were 0 μmol/l (95% confidence intervals contain only zero for both rosehip treatments). There were significant differences in AUC, maximal paracetamol concentration and in time to maximal paracetamol concentration. The median maximal paracetamol concentration was 147 μmol/l for water, which is significantly higher than the median for rosehip drink with Lp299v,113.5 μmol/l, and than the median for rosehip-drink without Lp299v, 106.5 μmol/l (p = 0.002, and p = 0.003); there were no significant difference between rosehip drink with or without Lp299v (p > 0.3).

**Conclusion:**

We have demonstrated an interaction between the uptake of paracetamol and the solvent in rosehip drink/Provia which mainly consists of long chain carbohydrates. This may in the clinic translate to the use of more drug than it is necessary.

## Background

Paracetamol is considered as a safe alternative to non-steroid anti-inflammatory drugs (NSAIDs) for the relief of mild to moderate pain in elderly patients, in patients with kidney disease, hypertensive and congestive heart failure subjects. In such patients NSAIDs may worsen the renal and cardiovascular function [1]. In addition, paracetamol does not cause gastrointestinal damage. However, elderly subjects often suffer from physical immobility in particular during hospital stays and in such conditions it has been observed that probiotics may be of great help.

*Lactobacillus plantarum *299v has the ability to survive the passage through the gastrointestinal tract and binds to cells of the intestinal epithelium in healthy individuals [2,3] and in critically ill subjects that are treated with antibiotics [4]. In patients with recurrent *Clostridium difficile*-associated diarrhoea, treatment with *Lactobacillus plantarum *299v in combination with metronidazole, reduced the recurrence of clinical symptoms in comparison with treatment with only metronidazole [5]. *Lactobacillus plantarum *299v can also reduce gas problems and pain [6] in people who suffer from irritable bowel syndrome. The symptoms are alternating diarrhoea and constipation, gases and pain. Intake of *Lactobacillus plantarum *(Lp) 299v can counteract certain unwanted bacteria in the intestine [3], and to have an anti-inflammatory effect by reducing the content of fibrinogen, reactive oxygen species and interleukin IL-6 in the serum of subjects in a proinflammative state [7]. It is widely used in Scandinavian countries, and for example regularly given to elderly patients with multidiseases in hospitals. The bacteria are usually given as a probiotic fruit drink (Provia). However, we observed that patients that vomit throw up undissolved tablets that have been taken together with the probiotic fruit drink several hours earlier. It seems therefore that the probiotic fruit drink might affect the solubility of the tablet, and that the absorption of the drug may be delayed. Based on results from a pilot in vitro study a human trial was performed where the concentration of paracetamol was measured in healthy volunteers after intake of water, rosehip drink or rosehip drink with Lp 299v.

## Findings

**Figure 1:**
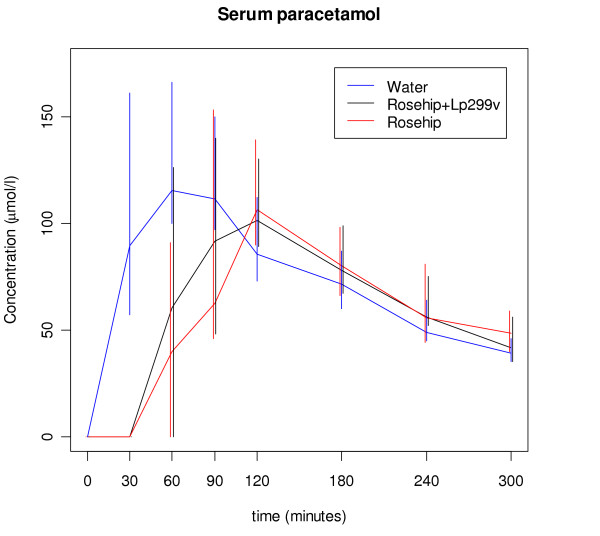
**Median of concentration of paracetamol in blood serum**. Error bars represent 95% confidence intervals for the median based on the measurements at that point.

There were large individual variations in the plasma levels of paracetamol, however, the general pattern was that most subjects had a larger AUC, a higher maximum level and shorter time to maximum when the tablets were ingested with water. The concentration of paracetamol in plasma increased rapidly when the paracetamol-containing tablets were consumed together with water and after 30 minutes a median level of 90 μmol/l was reached (Figure 1), with a 95% confidence interval of (57,161). The corresponding 30 minutes values of paracetamol levels in the presence of rosehip with or without Lp299v were 0 μmol/l where the 95% confidence intervals contain only zero for both rosehip treatments. There were significant differences in AUC, maximal paracetamol concentration and in time to maximal paracetamol concentration (p < 0.001 for AUC, p = 0.014 for maximum concentration and p < 0.001 for time to maximum according to Friedman's test). The median maximal paracetamol concentration was 147 μmol/l for water, which was significantly higher than the median for rosehip drink with Lp299v,113.5 μmol/l, and than the median for rosehip-drink without Lp299v, 106.5 μmol/l (p = 0.002, and p = 0.003) (Table 1), but there were no significant differences between rosehip drink with or without Lp299v (p > 0.3). The AUC values also differed (Table 1, p < 0.001, Friedman's test) and this was mainly because the rosehip drinks gave a lower paracetamol concentration up to 90 minutes after the start, whereas the paracetamol concentrations were similar for the three treatments in the interval 120–300 minutes (Figure 1). Post hoc tests showed significant differences between water and rosehip drink (p = 0.001), water and rosehip drink with Lp299v (p < 0.001), but no difference between rosehip drinks with or without Lp299v (p > 0.3).

**Table 1:** Statistical analysis of the paracetamol concentration in the three studied groups. Values are given as median and 95% confidence intervals (CI).

Variable	Water	Rosehip	Provia
Max plasma concentration (μmol/l)	147 (median) 117–193 (CI)	106 98–153	114 93–148

AUC (μmol min/l)	22000 19200–27400	18400 14900–21700	18600 14800–24900

Max time (min)	60 30–90	120 120–120	90 90–120

## Discussion

The maximal plasma concentration after intake of paracetamol tablets together with water is normally reached after 0.5 to 1 hour [8] but there are many factors that can modify its uptake [1]. In the present study the mean paracetamol plasma concentration was highest after 1 hour for water (Table 1), but there were large individual differences and the maximal plasma concentration was reached earlier for six subjects (0.5 hour) and much later for two subjects (2 hours). The time to maximum was not significantly different between the two rosehip drinks (w/wo Lp299v, p > 0.3) but there were significant differences between these and water. The mean time to maximum concentration was 65 minutes for water, 100 minutes for Provia and 110 minutes for rosehip drink. The median times with 95% confidence intervals are reported in Table 1 (for the median).

Intake of rosehip together with the tablets gave a lower paracetamol concentration in the plasma, especially in the time period up to 90 minutes; see AUC (Table 1). However, there were no differences whether Lp299v was present or not, and the probiotic bacteria thus presumably do not affect the uptake of paracetamol.

Factors that can influence the uptake of a drug when it is taken together with food include changes in dissolution, binding to food components and effects on gastric motility and emptying. An earlier study has shown that if a standardised breakfast is taken together with two tablets containing in total 650 mg paracetamol, the maximal plasma concentration was decreased by 38% when compared with tablets taken with water [8]. In the present study the maximal plasma concentration was decreased by 25% when the tablets were taken together with rosehip instead of water. In the study by Divoll et al. [8] the breakfast probably affected the gastric emptying, a factor that is minor in the present study since a low fat meal consisting only of a beverage was served. We believe that the main factor for the lower uptake of paracetamol in our study is effects on solubility that was also shown in a preliminary *in vitro *study (unpublished). In this study we found that paracetamol tablets immersed in rosehip drink with or without Lp299v do not dissolve during an observation period of > 4 hrs whereas the tablets are fully dissolved in water within 10 minutes (a well known fact).

What is it in the rosehip drink that makes this difference? We hypothesize that the carbohydrate content in standard fruit drinks can form a sheath around the tablets which prevents their dissolution. The structure of the fruit drinks consists mainly of carbohydrates and these may form a thick viscous layer consisting of long carbohydrate chains which can not easily be broken down and enwraps the tablets. In support, Elias [9] demonstrated that the presence of mono- and disaccharides reduced the rate of gastric emptying and the magnitude was proportional to the concentration of carbohydrates. It was hypothesised that this was due to the stimulation of duodenal osmoreceptors as based on infusion of glucose into the duodenum which suppressed the gastric emptying [10]. Furthermore, Costill and Saltin [11] noted a progressive decrease in the rate of gastric emptying with increases in the glucose concentration of a test meal. In addition, others observed a decrease in the rate of gastric emptying at increasing carbohydrate concentrations in different drinks [12,13]. More recently Murray and colleagues [14] found that repeated ingestion of 8% carbohydrate containing beverages reduced the gastric emptying rate, whereas lower concentrations did not.

We have presently revealed another aspect of carbohydrate concentration in beverages; it may modify the dissolution of paracetamol tablets. As we have shown it affects the possibility of the paracetamol containing tablets from dissolution and thus forms the difference with the diffusivity of the tablet to dissolve in water. We have in preliminary studies *in vitro *examined different types of tablets containing paracetamol and the tablets behave the same way irrespective of brand (unpublished), thus it is due to the interaction between the tablets with the solvent that makes the difference.

Food can affect the onset and intensity of a single dose but it is often a mixture of different ingredients [1]. Here we have demonstrated an interaction between the uptake of paracetamol and the solvent in rosehip drink/Provia which mainly consists of long chain carbohydrates.

The clinical consequences of this finding may be that we use more medication to our elderly that their condition requires. Probiotics are useful but we might have to choose wisely the vehicle for their administration, particularly to the elderly.

## Methods

### Subjects

Nineteen healthy subjects (8 men and 11 women) were included. Before inclusion, their health status was checked by measurement of the blood pressure, pulse rate, blood sodium, potassium, creatinine (kidney function), bilirubin, ALP, ASAT, ALAT, GT (liver function) and haemoglobin using accredited methods at the department of Clinical Chemistry, University Hospital, Lund, Sweden.

One woman was difficult to sample blood from and her participation was terminated. Of the eighteen subjects that finished the study, the age span was 20–45 (mean 26) years, and the mean body mass index was 23. The subjects were not allowed to take drugs but for contraceptives. Smoking or the use of snuff was also not allowed and they should have normal eating habits. The subjects were given both oral and written information about the study and they gave a written consent to participate in the study. The study was approved by the Local Ethics Committee in Lund (783/2005) and by the Medical Products Agency.

### Design

The subjects came in the morning to the clinical research unit after fasting for six hours. The subjects were served the test products, 3 tablets each containing 500 mg paracetamol (Panodil, GlaxoSmithKline) together with either rosehip drink, rosehip drink with Lp299v or water (200 ml of each drink). Each subject was given all test drinks, with 2–3 week in between the drinks. The order of the drinks was randomised and blinded to the staff involved.

Blood samples for paracetamol analysis were withdrawn immediately before the test meal (time 0) and then at every 30-min interval until 90 min, thereafter a one hour interval was used until five hours was reached. The blood was withdrawn via an indwelling canula inserted into an antecubital vein.

### Blood analysis

Plasma was separated from the blood and paracetamol was analysed with an enzyme multiplied immuno test (Hitachi Modular-P instrument) using an accredited method at the department of Clinical Chemistry, Lund.

### Statistical methods

For each subject and test meal, the area under the curve of the plasma concentration of paracetamol as a function of time was calculated (AUC) using the trapezoidal rule. The differences between the AUC and the maximal plasma concentration for each test were evaluated with Friedman's test. If a significant difference was found, the test was followed by pair wise comparison using Wilcoxon signed rank test. The statistical calculations were performed with SPSS, version 15.0. The alternative hypothesis is considered statistically significant when the probability of the null is 0.05 or lower.

## Competing interests

The authors declare that they have no competing interests.

## Authors' contributions

All authors read and approved the manuscript. UÅ and LE designed the Research project and carried it out together. LE was most responsible for writing the final manuscript.
